# A Giant Right Atrial Hemangioma- Case Report

**DOI:** 10.7759/cureus.24622

**Published:** 2022-04-30

**Authors:** Rajeev Thilak, Ananthkumar Sivanesan, Hemachandren Munuswamy, Pampa Ch Toi

**Affiliations:** 1 Cardiothoracic Surgery, Jawaharlal Institute of Postgraduate Medical Education & Research, Puducherry, IND; 2 Cardiovascular Surgery, Jawaharlal Institute of Postgraduate Medical Education & Research, Puducherry, IND; 3 Pathology, Jawaharlal Institute of Postgraduate Medical Education & Research, Puducherry, IND

**Keywords:** benign tumors, adult cardiac surgery, cavernous, hemangioma, cardiac tumor in adults

## Abstract

A primary cardiac tumor is sporadic. Most cardiac tumors are benign, with cardiac myxoma being the most common tumor. The incidence of cardiac hemangioma is extremely low. We report a 55-year-old female patient admitted for chest pain and breathlessness and, on evaluation by Echocardiography and Computed tomography, was diagnosed with a right atrial mass. The patient was taken up for surgery. The excised right atrial mass was confirmed as atrial hemangioma by postoperative histopathology. Cardiac hemangioma should be suspected when imaging shows a homogenous mass with vascularity. We present this case as the tumor is sporadic and illustrate the technical difficulties we encountered during the surgery.

## Introduction

Cardiac hemangiomas are rare benign tumors, and their incidence is around 3% of all cardiac tumors [1]. Most cardiac hemangiomas are asymptomatic, and they grow at a prolonged rate and usually do not metastasize. However, there are case reports of hemangiomas invading the conductive system. It can be dangerous as there is a risk of life-threatening complications such as syncope, stroke, and sudden cardiac death [2]. Surgical resection offers a complete cure, and patients have a good prognosis after complete resection. Since the tumor is rare, there are no clear indications and operative methods for cardiac hemangiomas. We present the technical challenges that we faced while operating this rare tumor and complete resection was possible though it was huge.

## Case presentation

**Figure 1:**
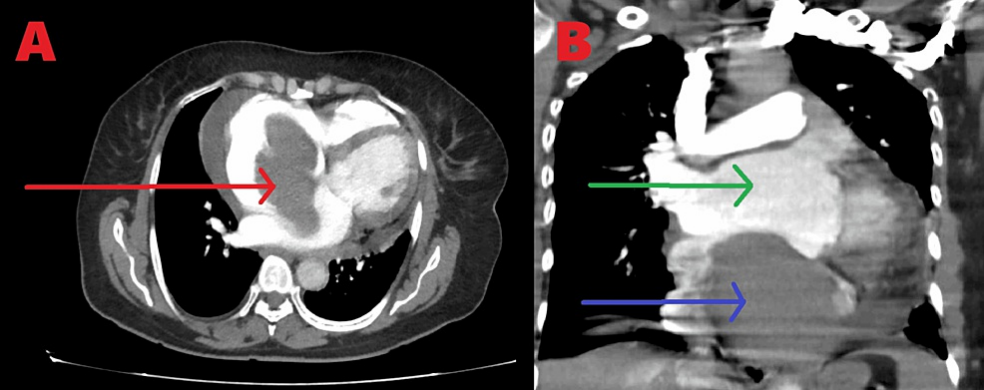
Computed Tomography; A: Red arrow showing a hypodense lesion arising from the atrial septum. B: Green arrow showing the free left atrium and the blue arrow showing the mass in the right atrium.

A 55-year-old female patient reported to us with chief chest pain and breathlessness complaints. She was evaluated and was found to have right atrial mass by Transthoracic Echocardiography. It revealed a homogenous, non-pedunculated mass arising from the right atrial septum. The tricuspid valve was free from the tumor. All valves were normal and biventricular function was normal. A coronary angiogram showed normal coronaries. Cardiac Computed Tomography (CT) was done, which showed a large hypodense lesion of size 113x77x71 mm in the right atrium arising from the atrial septum, and the mass was partially occluding the Inferior Vena Cava (IVC) (Figure 1). 

**Figure 2:**
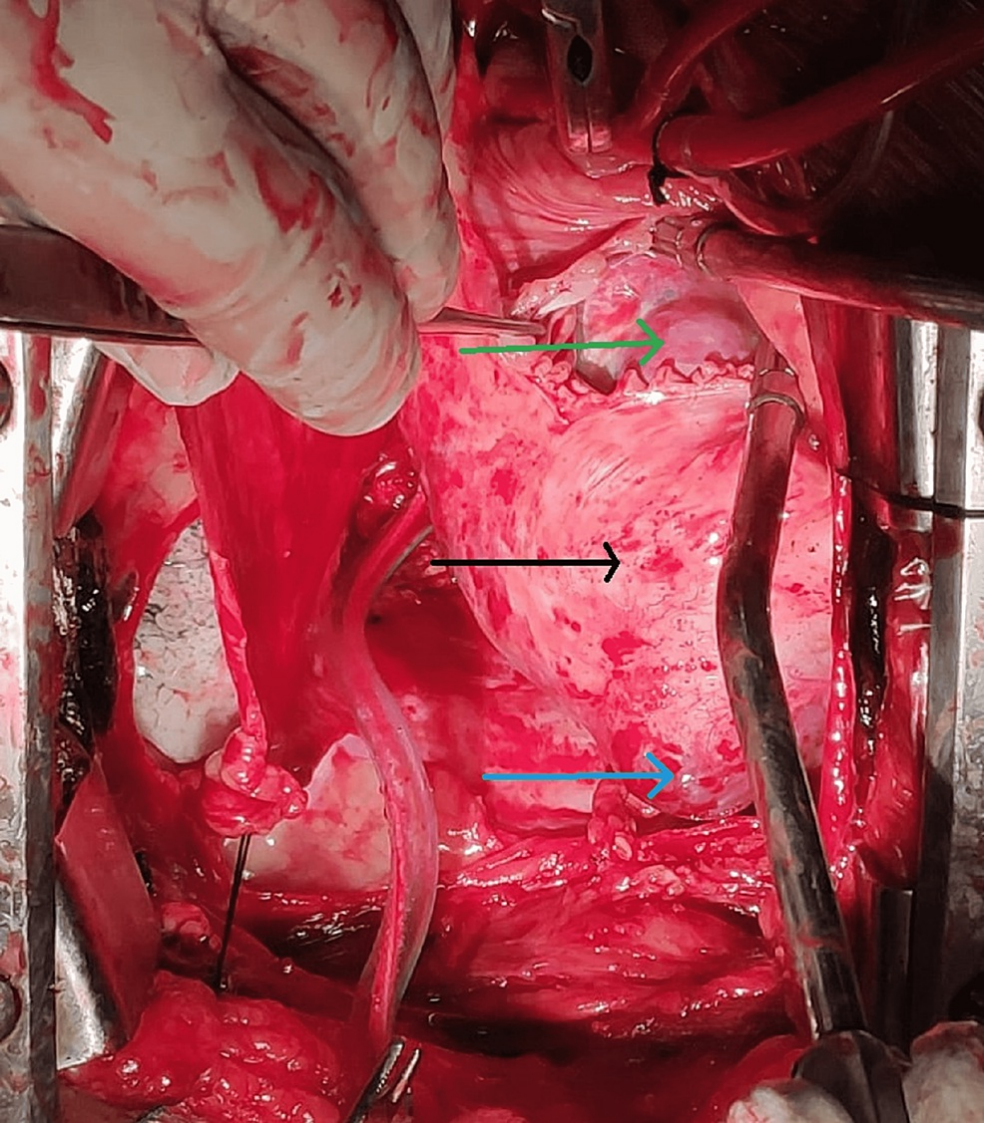
Intra-operative image; green arrow showing right atrial tumor, black arrow showing the stretched right atrial wall, and the blue arrow showing the right atrial involvement by the tumor

**Figure 3:**
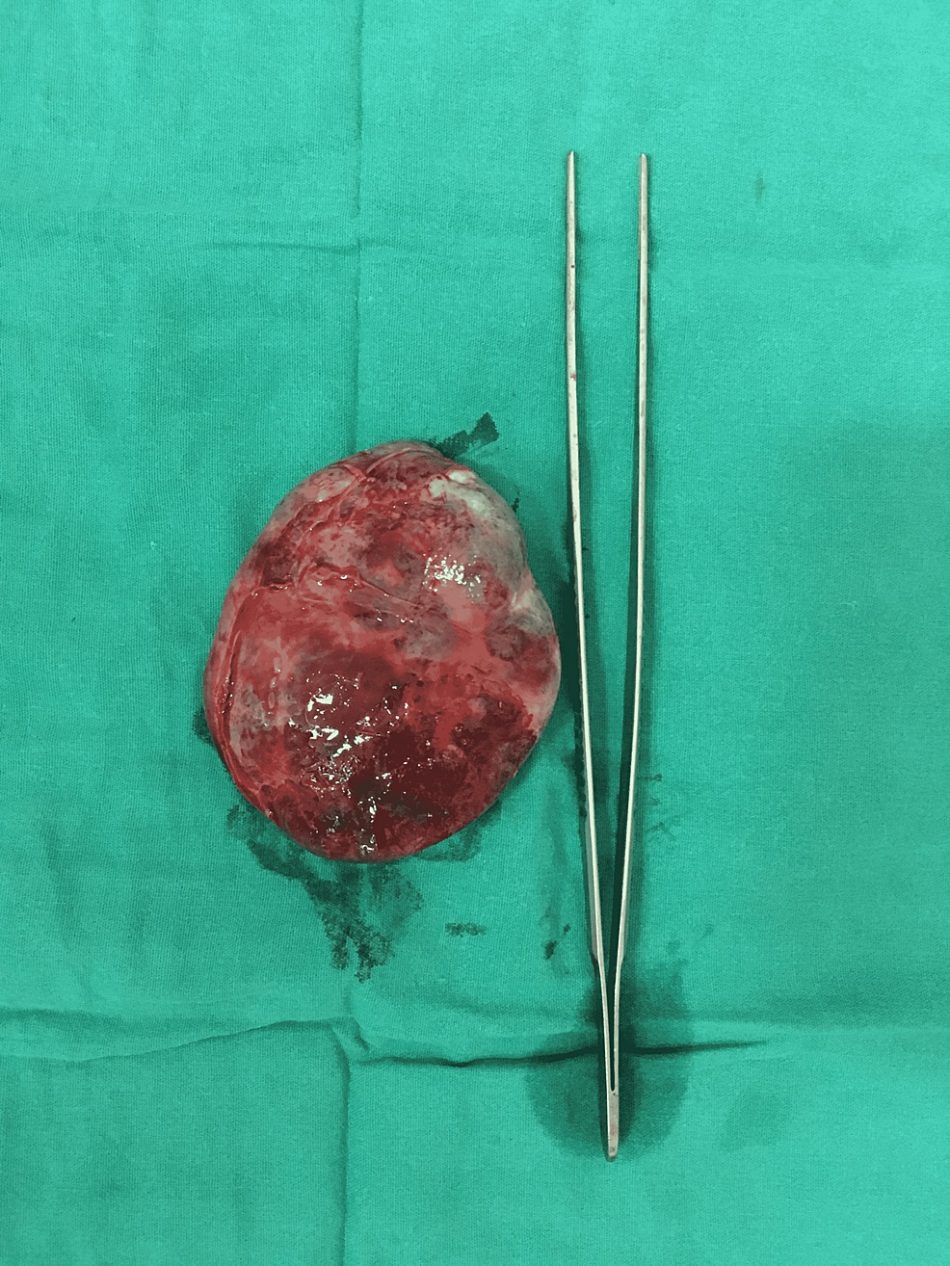
Specimen after surgical resection of the tumor.

**Figure 4:**
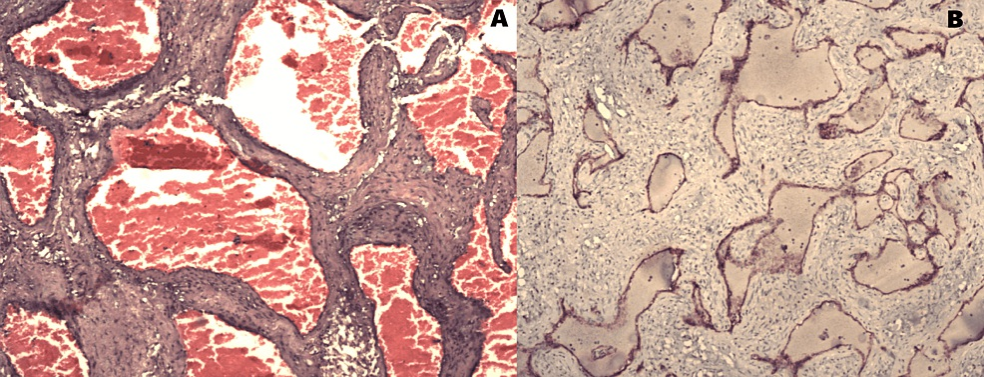
A. Histopathology slide (Hemotoxylin and eosin stain, 4X magnification) showing dilated vascular channels; B: Immunohistochemistry slide (CD 34 stain, 4X magnification) showing high-lightened endothelial cells.

The patient was planned for surgical excision of the tumor. The chest was opened. Since the tumor was partially occluding the IVC opening into the right atrium, Cardio-pulmonary bypass was established with Aorta, superior vena cava (SVC), and right femoral vein cannulation. There was a mild difficulty looping the IVC due to the large size of the tumor stretching between IVC joining the right atrium and atrioventricular groove. So IVC looping was done after establishing the bypass. Superior Vena Cava (SVC) was snared, and IVC was snared below the tumor. The heart was arrested with Del Nido cardioplegia, and the right atrium (RA) was opened. A large right atrial mass arose from the atrial septum and splayed the coronary sinus and the IVC (Figure 2). The involved and stretched-out part of the right atrial wall was excised along with the tumor (Figure 3 ). RA was reconstructed with a pericardial patch between the IVC and the coronary sinus. The patient did not have any post-operative complications. Post-operative ECG was normal. An echocardiogram showed normal tricuspid and mitral valves, and there was no residual mass in the right atrium. The histopathology was reported as a cavernous hemangioma (Figures 4). The patient was followed up after one month. She was asymptomatic, and Echocardiogram showed no residual mass or any recurrence.

 

## Discussion

Cardiac hemangiomas can arise in the pericardium, myocardium, or endocardium. Endocardial tumors are common and can occur at any age and arise in any heart chamber. They are more commonly found in the right atrium, as in our case. The left atrium is the second most common chamber involved [1]. Hochberg and Robinson were the first to do cardiac hemangioma excision in 1950 [3]. They occur due to abnormal hyperplasia or dilation of small arterioles, venules, or capillaries. These tumors are classified into four types based on their histological appearance. They are cavernous, capillary, arteriovenous, and mixed types. Cavernous hemangioma is the most common type of cardiac hemangioma [4].

Most of the patients are asymptomatic, and they usually manifest when there is a hemodynamic compromise due to their mass effect. Clinical features depend on the patient's age, the size of the tumor and its growth rate, and myocardial or pericardial infiltration of the tumor. In a few cases, they can cause compression of the esophagus and trachea or may cause ventricular prolapse. Though the tumor size was significant in our patient, there was no compression of any structures as the tumor's growth was outward, stretching the right atrial wall [5]. Our patient presented with chest pain and breathlessness, which can be attributed to the large size of the tumor and partial occlusion of the IVC at the Ostia.

Myxomas appear as a lobulated spherical or oval mass with high mobility [1]. It can be ruled out in our case as the mass was homogenous and non-pedunculated in echocardiogram and Computed tomography (CT). A cardiac lipoma is a closer differential diagnosis to hemangioma and has similar radiological features [6]. Tumor blush is mainly seen in hemangioma, which can help to differentiate from lipoma. Cardiac hemangiosarcoma is a malignant tumor that has a similar presentation to hemangioma. Histopathological examination is essential to differentiate them.

Echocardiography is the most critical screening and diagnostic tool. Hemangiomas will appear as a homogeneous solid mass, as in our case. CT scan will help in identifying the location of hemangioma, its size, and its extra-cardiac involvement. A conventional angiogram will show a tumor blush, one of the characteristic features of cardiac hemangioma [7]. 

Surgery is the definitive treatment for cardiac hemangioma. However, radiotherapy can be used in inoperable tumors [8]. Yoshikawa successfully utilized radiotherapy for treating atrial hemangioma in a 10-week-old infant [9]. Cardiac hemangiomas close to the conduction system and coronary arteries pose great danger while dissecting. In our case, the tumor was close to the conduction bundle, but we could excise the tumor without damaging the nerve bundle. Endocardial tumors are well-circumscribed soft masses. Surgery should be done mainly to relieve the symptoms. It is vital to make a pathological diagnosis to rule out malignant tumors and reduce the potential risk of embolization and rupture, leading to sudden death. Complication following surgery occurs when the vessel feeding the mass is not ligated properly. The common complications are valve regurgitation, conduction disturbances, and cardiac fistula, which are very rare [10].

## Conclusions

Cardiac hemangioma is rare and should be kept as a differential diagnosis when imaging shows a homogenous mass with vascularity. Careful preoperative evaluation should be made, which helps in surgical planning. Preoperative computed tomography imaging should be done to identify the extent of the tumor. An echocardiogram is mandatory in pre and post-operative periods to look for the residual mass and valvular regurgitation. Surgical excision of hemangioma has the best prognosis.
